# Explainable AI for Chronic Kidney Disease Prediction in Medical IoT: Integrating GANs and Few-Shot Learning

**DOI:** 10.3390/bioengineering12040356

**Published:** 2025-03-29

**Authors:** Nermeen Gamal Rezk, Samah Alshathri, Amged Sayed, Ezz El-Din Hemdan

**Affiliations:** 1 Department of Computer and Systems Engineering, Faculty of Engineering, Kafrelsheikh University, Kafrelsheikh 33516, Egypt; nermeen_rezk@eng.kfs.edu.eg; 2 Department of Information Technology, College of Computer and Information Sciences, Princess Nourah bint Abdulrahman University, P.O. Box 84428, Riyadh 11671, Saudi Arabia; 3 Department of Electrical Energy Engineering, College of Engineering & Technology, Arab Academy for Science Technology & Maritime Transport, Smart Village Campus, Giza 12577, Egypt; 4 Industrial Electronics and Control Engineering Department, Faculty of Electronic Engineering, Menoufia University, Menoufia 32952, Egypt; 5 Department of Computer Science and Engineering, Faculty of Electronic Engineering, Menoufia University, Menoufia 32952, Egypt; ezzeldinhemdan@el-eng.menofia.edu.eg; 6 Structure and Materials Research Lab, Prince Sultan University, Riyadh 12435, Saudi Arabia

**Keywords:** chronic kidney disease prediction (CKD), medical Internet of Things (MIoT), explainable machine learning (XAI), few-shot learning, generative adversarial networks (GANs), SHapley Additive exPlanations (SHAP), local interpretable model-agnostic explanations (LIME)

## Abstract

According to recent global public health studies, chronic kidney disease (CKD) is becoming more and more recognized as a serious health risk as many people are suffering from this disease. Machine learning techniques have demonstrated high efficiency in identifying CKD, but their opaque decision-making processes limit their adoption in clinical settings. To address this, this study employs a generative adversarial network (GAN) to handle missing values in CKD datasets and utilizes few-shot learning techniques, such as prototypical networks and model-agnostic meta-learning (MAML), combined with explainable machine learning to predict CKD. Additionally, traditional machine learning models, including support vector machines (SVM), logistic regression (LR), decision trees (DT), random forests (RF), and voting ensemble learning (VEL), are applied for comparison. To unravel the “black box” nature of machine learning predictions, various techniques of explainable AI, such as SHapley Additive exPlanations (SHAP) and local interpretable model-agnostic explanations (LIME), are applied to understand the predictions made by the model, thereby contributing to the decision-making process and identifying significant parameters in the diagnosis of CKD. Model performance is evaluated using predefined metrics, and the results indicate that few-shot learning models integrated with GANs significantly outperform traditional machine learning techniques. Prototypical networks with GANs achieve the highest accuracy of 99.99%, while MAML reaches 99.92%. Furthermore, prototypical networks attain F1-score, recall, precision, and Matthews correlation coefficient (MCC) values of 99.89%, 99.9%, 99.9%, and 100%, respectively, on the raw dataset. As a result, the experimental results clearly demonstrate the effectiveness of the suggested method, offering a reliable and trustworthy model to classify CKD. This framework supports the objectives of the Medical Internet of Things (MIoT) by enhancing smart medical applications and services, enabling accurate prediction and detection of CKD, and facilitating optimal medical decision making.

## 1. Introduction

More than 700 million individuals worldwide suffer from chronic kidney disease (CKD), which affects 10% of the world’s population [1]. According to global public health studies, 78% of individuals with chronic kidney disease (CKD) live in low- and middle-income countries, where health systems face challenges due to systemic injustices and a lack of resources. Without functioning kidneys, the average survival time is only about 18 days, making dialysis and kidney transplants highly necessary. CKD is classified into different stages: stage 1/2 is considered early-stage kidney disease, stage 3/4 is considered mid-stage kidney failure, and stage 5 renal failure is considered late-stage kidney disease. Therefore, early detection of individuals at risk for developing kidney failure offers a chance to implement focused interventions to change the course of the disease. However, the diagnosis of CKD requires testing urine for protein levels and blood for measuring biochemical kidney function. The results from these tests are helpful in classifying CKD from Stage 1 (minimal damage) to Stage 5 (kidney failure) [2].

Even though these studies are straightforward, proteinuria data is often missing, compromising the ability to provide optimal therapy. Additionally, these two tests do not consider numerous variables that could influence disease progression. Currently, clinical judgment, supported by the course of kidney disease, is used to predict who is likely to develop kidney failure. Clinicians must be able to identify patients who are at risk for progressive illness and renal failure by integrating other relevant data sets [3]. To facilitate early detection, a variety of statistical predictors have been developed. A person with chronic kidney disease (CKD) has a probability of developing kidney failure between the ages of two and five, according to a risk prediction method known as the kidney failure risk equation (KFRE) [4]. The key factors in this calculation are age, sex, eGFR, and the urine albumin/creatinine ratio; on the other side, the adjusted calcium, phosphorus, bicarbonate, and serum albumin are optional measures. The equation’s major limitations are that it only accounts for renal failure (requiring dialysis) as an outcome, only applies to later stages of CKD (G3–G5), and requires a urine albumin/creatinine ratio, which is rarely done in clinical practice. Furthermore, the KFRE is a static equation that disregards several readings and changes in variables throughout time [5].

**Figure 1:**
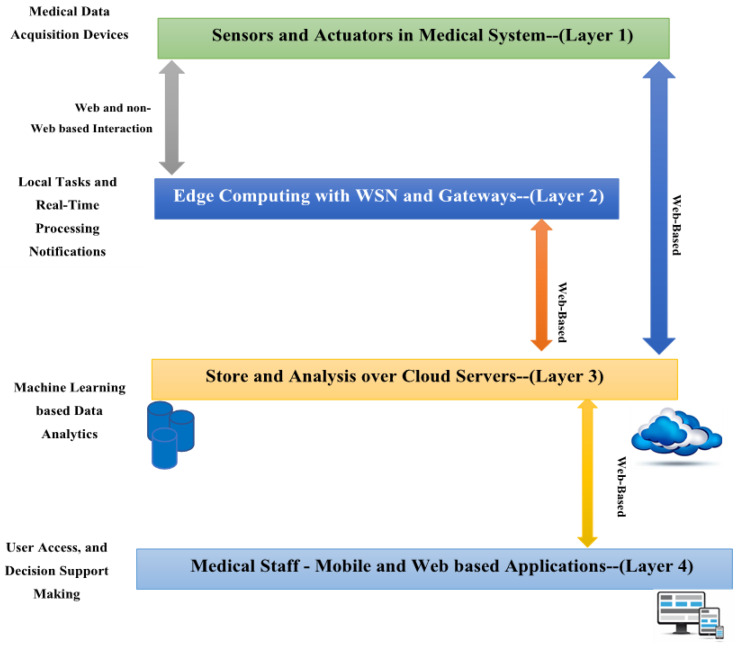
A standard medical IoT (MIoT) model [10].

Artificial intelligence (AI) has many applications nowadays in various fields such as healthcare, environment, and transportation [6,7]. In biomedical applications, artificial intelligence (AI) is now a major force behind individualized diagnosis, treatment planning, and illness management. The incorporation of Internet of Things (IoT) technologies further advances this development. Advancements in artificial intelligence (AI) and machine learning (ML) offer significant potential for bioscience and improving disease classification and prediction in biomedical applications [8,9]. ML methods, for example, can support the early detection of organ conditions with exceptional accuracy and dependability. For instance, ML techniques can reliably and accurately assist in the early identification of organ diseases. Medical Internet of Things (MIoT) applications have been enhanced by the recent emergence of IoT and medical systems as complementing technologies. Incorporating Internet of Things (IoT) technology into healthcare to provide effective, real-time patient monitoring, diagnosis, and management is known as the Medical Internet of Things (MIoT). Real-time monitoring, analysis, and decision making in complicated healthcare contexts are made easier by connecting healthcare services with IoT devices, which significantly improves patient care. The MIoT system consists of multiple layers, as shown in Figure 1 below:

- Layer 1: This layer is driven by MIoT sensors and actuators, which are responsible for collecting and monitoring various medical and healthcare data.
- Layer 2: This layer involves the use of gateway and edge devices, which serve as intermediaries linking the wireless sensor network (WSN) to cloud servers. These devices are a key part of the MIoT-based control platform.
- Layer 3: Cloud computing is utilized in this layer, showcasing its ability to power intelligent medical IoT systems. The proposed system uses cloud servers to store and process data from the industrial control field, collected through the MIoT-based controller. The medical data gathered is periodically transmitted to the relevant channel via an IoT protocol like Constrained Application Protocol (CoAP).
- Layer 4: This layer focuses on developing mobile and web applications that interface with cloud servers to retrieve analytical results generated by applying machine learning techniques to stored medical and healthcare data. The goal is to provide actionable insights to support decision making within healthcare institutions.

Overall, the multi-tier architecture of an MIoT system enables smooth data flow, storage, and subsequent analysis.

Clinical decision making can be improved with the help of artificial intelligence (AI), especially machine learning (ML) models, which help in analyzing various biomedical signal [8]. Clinical and biological data are routinely collected in large quantities. Because of this, it is recommended to integrate machine learning models that are intended to identify non-linear patterns in big, complicated datasets and forecast the behavior of future variables. Selecting which individual variables to have an impact in an ML model requires careful consideration because variable relevance significantly affects accuracy and data gathering [11]. However, the datasets in some studies suffer from missing values. So, by learning the underlying data distribution and producing believable values to fill in the gaps, GANs can be used to forecast missing values in datasets. Due to their poor interpretability, many of the earlier models for CKD prediction have a “black box” aspect that prevents clinical application. By enhancing model transparency and reliability, XAI approaches such as SHapley Additive exPlanations (SHAP) and Local Interpretable Model-agnostic Explanations (LIME) help close the gap between machine learning predictions and clinical applicability. A crucial tool that complements XAI methodologies is counterfactual analysis, which modifies input data points significantly to track changes in the model’s predictions and offer concrete and useful insights into the model’s decision-making process. We created an explainable longitudinal machine learning model that could successfully detect CKD patients [12].

This paper presents an explainable AI framework for chronic kidney disease prediction in medical IoT by integrating GAN-based data imputation with few-shot learning for accurate and interpretable classification. Therefore, the objectives that follow are attempted to be accomplished by this comprehensive study:

- This procedure improves the model’s functionality and offers insightful information about the main causes of CKD. For the modeling, we employed support vector machines (SVM), logistic regression (LR), decision trees (DT), random forests (RF), and Voting Ensemble Learning (VEL). A few-shot learning techniques like prototypical networks and model-agnostic meta-learning were also used and integrated with the XAI to explain why a person is likely to have CKD or not.
- A proposed preprocessing method such as generative adversarial networks (GANs) has been implemented to deal with missing values in datasets, yielding superior results by integrating tried-and-tested practices compared to existing techniques.
- Accurate CKD prediction and clear explanation are made possible by combining machine learning and XAI approaches.
- It is shown the superior performance of our proposed model in terms of accuracy, precision, recall, score, ROC curve, interpretability, and resilience by comparing its performance with that of state-of-the-art models that had previously been applied to the same dataset.
- The proposed model is specifically designed for real-world medical IoT (MIoT) applications, enhancing the intelligent prediction and monitoring of CKD through its scalability and flexibility. Its structured approach enables various MIoT components to function efficiently, regardless of the deployment environment.

The rest of this paper is organized as follows: Section 2 provides a concise overview of pertinent literature in the paper’s subject area. Section 3 explains the material and the methods used in this study. Section 4 presents the proposed methodology for CKD prediction, while Section 5 shows the results and outcomes analysis of the proposed framework. Finally, the paper’s conclusion and future scope are presented in Section 5.

## 2. Related Work

Recent advancements in deep learning have led to the development of more transparent and interpretable models for medical diagnosis. To address these issues, Tanim et al. [13] present DeepNetX2, a bespoke deep neural network that integrates explainable artificial intelligence (XAI) methods, particularly SHapley Additive exPlanations (SHAP) and local interpretable model-agnostic explanations (LIME). These methods increase the model’s decision-making transparency, which boosts the forecasts’ credibility. A thorough data pretreatment procedure using a tailored Spearman’s correlation coefficient feature selection strategy is part of the suggested methodology. Instead of oversimplifying to the point of losing efficiency, this preprocessing limits complexity to only pertinent elements that enhance efficacy. The PIMA dataset, the local private dataset, and the Type-2 diabetes dataset were used to thoroughly evaluate DeepNetX2.

The authors in [14] discuss the present and probably future uses of AI in the treatment of diabetes and its comorbidities, such as medication compliance, hypoglycemia diagnosis, diabetic neuropathy, diabetic kidney disease, diabetic eye disease, diabetic foot ulcers, and diabetic heart failure. The ability of artificial intelligence to manage sizable and intricate datasets from several sources makes it beneficial. The calculation gets more complicated and precise with each new kind of data added to a patient’s clinical picture. Emerging medical technologies are built on artificial intelligence, which will drive future improvements in patient health and diagnostic integrity as well as the diagnosis of diabetes complications. To improve dataset preparation for CKD classification and create a web-based application for CKD prediction.

A machine learning-based kidney disease prediction (ML-CKDP) model is created in [15]. A thorough data pretreatment procedure, numerical value conversion for categorical variables, missing data imputing, and normalization using min–max scaling are all part of the suggested model. Correlation, chi-square, variance threshold, recursive feature elimination, sequential forward selection, Lasso regression, and ridge regression are some of the methods used to refine the datasets during feature selection. Random forest (RF), AdaBoost (AdaB), gradient boosting (GB), boost (XgB), naive Bayes (NB), support vector machine (SVM), and decision tree (DT) are the seven classifiers used by the model to predict CKDs. The models’ efficacy is evaluated using accuracy measurements and confusion matrix statistics analysis, as well as computing the area under the curve (AUC), especially for positive case categorization. The 100% accuracy rate of random forest (RF) and AdaBoost (AdaB) is demonstrated using a variety of validation techniques, such as data splits of 70:30, 80:20, and K-Fold set to 10 and 15. Under various splitting ratios, RF and AdaB regularly achieve flawless AUC values of 100% across a variety of datasets. Naive Bayes (NB) is particularly effective; it has the shortest training and testing durations for all datasets and split ratios. To operate the model and improve accessibility for stakeholders and healthcare professionals.

To predict chronic kidney disease, Khan et al. [16] conduct a variety of machine learning models, including logistic regression, random forest, decision tree, k-nearest neighbor, and support vector machine with four kernel functions (linear, Laplacian, Bessel, and radial basis kernels). Records from a case-control study including patients with chronic renal disease in Pakistan make up the dataset that was used. A variety of performance metrics, such as accuracy, Brier score, sensitivity, Youden’s index, and F1-score, were calculated to compare the models’ classification and accuracy. To categorize patients into two groups: those who advanced to CKD stages 3–5 during follow-up (positive class) and those who did not (negative class). The authors in [17] created four machine learning algorithms: logistic regression, random forests, neural networks, and eXtreme gradient boosting (XGBoost). The model’s ability to distinguish between the two classes was assessed using the area under the receiver operating characteristic curve (AUC-ROC) for the classification test. The concordance index (C-index) and integrated Brier score were utilized for model evaluation, while Cox proportional hazards regression (COX) and random survival forests (RSFs) were utilized for survival analysis to forecast the progression of CKD. Additionally, the outcomes of the models were interpreted using restricted cubic splines, variable importance, and partial dependence plots.

A combination of the models was included in [18] half-and-half model. The Irregular Timberland classifier served as the meta classifier, while the basis classifiers used were XGBoost, arbitrary woods, strategic relapse, AdaBoost, and the crossover model classifiers. This analysis’s primary goal was to assess the top AI grouping strategies and select the most accurate classifier. This method achieved the highest level of accuracy and fixed the problem of overfitting. Precision was the primary focus of the evaluation, and we implemented a comprehensive analysis of the important writing in even configuration.

The authors employed four of the best AI models and developed a second model called “half and half”, using the UCI Persistent Kidney Disappointment dataset for predictive analysis. The “black box” aspect of conventional machine learning predictions was addressed in [2] by using explainable machine learning to predict CKD. The extreme gradient boost (XGB) machine learning method showed the highest accuracy out of the six that were assessed. SHapley Additive exPlanations (SHAP) and partial dependency plots (PDP), which clarify the reasoning behind the predictions and aid in decision making, were used in the study for interpretability. Additionally, a graphical user interface with explanations was created for the first time to diagnose the probability of chronic kidney disease. Explainable machine learning can help medical personnel make precise diagnoses and pinpoint the underlying causes of chronic kidney disease (CKD), which is a serious condition with high stakes.

In [19], twelve full-featured classification algorithms based on machine learning were employed. The synthetic minority over-sampling technique (SMOTE) was employed to address the class imbalance issue in the CKD dataset and evaluate the effectiveness of machine learning classification models using the K-fold cross-validation technique. Support Vector Machine, Random Forest, and Adaptive Boosting are the three classifiers with the highest accuracy that were chosen to employ the ensemble technique to enhance performance after the results of twelve classifiers with and without the SMOTE technique. While Qin et al. [20] focus on utilizing machine learning techniques to create a CKD predictive model by examining a dataset including 25 columns and 9993 rows that contain important kidney health data. For the best CKD prediction, several techniques are examined, including random forest, logistic regression, decision trees, support vector machines (SVM), k-nearest neighbors (KNN), and naive Bayes. Correcting missing data guarantees accurate results. The ultimate objective is to offer a dependable, reasonably priced model for early CKD detection, which will benefit patients and healthcare providers by facilitating prompt intervention and accelerating diagnosis.

In [21], the study begins with 25 variables; however, at the conclusion, it has reduced the list to 30% of those factors as the most effective subset for CKD identification. In a supervised learning setting, twelve distinct machine learning-based classifiers have been evaluated. Twelve different machine learning-based classifiers have been studied within the parameters of a supervised learning environment. The XGBoost classifier has the best performance metrics. The study’s methodology leads to the conclusion that modern advances in machine learning, combined with predictive modeling, offer an intriguing means of discovering new. To increase the accuracy of CKD prediction, the authors in [22] suggested a hybrid convolutional neural network (CNN) support vector machine (SVM) model. Performance was enhanced by combining SVM for classification with CNN for feature extraction. SMOTE was used for a large clinical dataset that included ten medical indicators in total.

A thorough evaluation of the literature on chronic renal disorders has been conducted. Table 1 provides a description of the literature review summary. The influence of variable selection and dataset features on model performance has been highlighted in earlier research on CKD prediction and prognosis using machine learning approaches. However, external validation on independent datasets is often lacking in these studies, which is important for evaluating generalizability. and ignoring missing values is also considered a critical step in improving CKD prediction. First, we use GANs to predict missing values in datasets where GANs can learn the underlying data distribution and generate realistic data samples. Secondly, due to the machine learning’s poor interpretability, many of the earlier models for CKD prediction have a “black box” aspect that prevents clinical application. By enhancing model transparency and reliability, XAI approaches such as SHapley Additive exPlanations (SHAP) and local interpretable model-agnostic explanations (LIME) help close the gap between machine learning predictions and clinical applicability. A crucial tool that complements XAI methodologies is counterfactual analysis, which modifies input data points significantly to track changes in the model’s predictions and offer concrete and useful insights into the model’s decision-making process. We created an explainable longitudinal machine learning model that could successfully detect CKD patients who would eventually develop kidney failure or not to overcome the shortcomings of earlier research.

## 3. Material and Methods

### 3.1. Generative Adversarial Networks (GANs)

**Figure 2:**
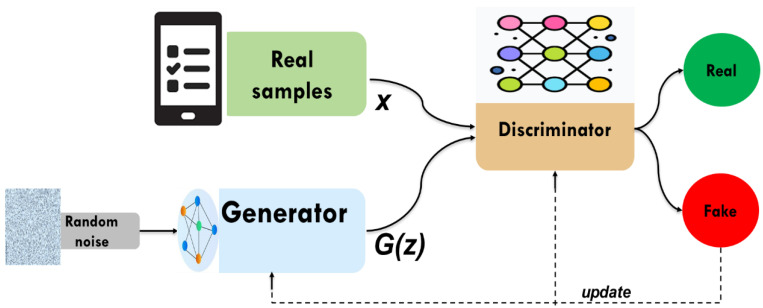
The structure of GAN.

Generative adversarial networks (GANs) are a class of artificial intelligence models designed to generate new data that resembles a given dataset. Introduced by Ian Goodfellow and his colleagues in 2014 [23]. GANs comprise a generator and a discriminator, both trained under the adversarial learning idea. The goal of GANs is to estimate the potential distribution of real data samples and generate new samples from that distribution. In data imputation, the generator attempts to predict missing values by learning the underlying data distribution. While the discriminator is often a binary classifier to evaluate whether the imputed values are real (from the original dataset) or fake (produced by the generator). The generator continuously improves its ability to generate realistic imputations through iterations, making GANs particularly powerful for analyzing data in the case of high dimensionality and complexity. GANs learn complex patterns from the data, which, unlike simpler imputation techniques like mean imputation or regression-based methods, provide more accurate and context-aware imputations. This adversarial process will continue as depicted in Figure 2. The structure of currently widely used deep neural networks can be used by both the discriminator and the generator. The optimization process of GANs is a minimax game process, and the optimization goal is to reach Nash equilibrium, where the generator is considered to have captured the distribution of real samples.

### 3.2. Few Learning Technique

**Figure 3:**
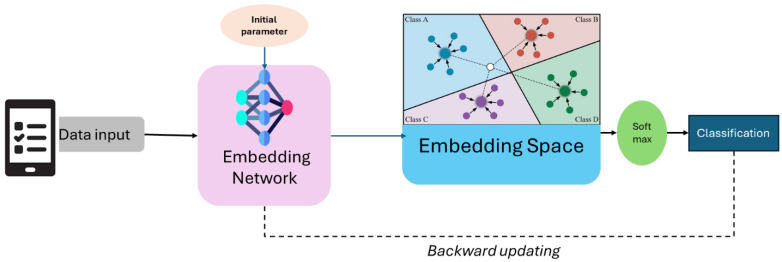
The framework of prototypical networks few-shot learning classification.

Few-shot learning techniques, such as prototypical networks and model-agnostic meta-learning (MAML), enable models to generalize effectively by leveraging prior knowledge, learning robust representations, or adapting quickly to new tasks. Few-shot learning is a machine learning paradigm that focuses on training models to be a powerful paradigm that empowers models to rapidly adapt and generalize to new tasks with minimal training examples [24]. As shown in Figure 3, prototypical networks are a metric-based approach that leverages the concept of class prototypes to classify new examples. During training, the network computes a prototype (mean representation) for each class in the support set (a small, labeled dataset). This figure depicts the few-shot learning application of prototypical networks in predicting chronic kidney disease (CKD). Patient data, which include clinical information and lab test results, are passed through an embedding network, extracting relevant features and mapping them into a lower-dimensional embedding space. In this space, different CKD stages cluster the data points, each represented as a prototype (centroid of known samples). The classification of a new test sample is done based on its proximity to these prototypes, using a distance metric such as the Euclidean distance. Finally, a softmax function assigns the sample to the most likely CKD stage. This approach enables accurate prediction with limited labeled data. These networks are particularly efficient because they rely on a single forward pass for inference, avoiding the need for complex optimization during testing.

On the other hand, model-agnostic meta-learning (MAML) is a meta-learning technique designed to enable models to quickly adapt to new tasks with minimal data. MAML works by learning a good initialization for the model’s parameters during meta-training, such that only a few gradient steps are needed to fine-tune the model on a new task during meta-testing. Unlike prototypical networks, which focus on learning a metric space, MAML is a general-purpose optimization-based approach that can be applied to any model trained with gradient descent. This flexibility makes MAML suitable for a wide range of applications. Both prototypical networks and MAML have significantly advanced the field of few-shot learning, each with its unique strengths.

### 3.3. Explainable Artificial Intelligence (XAI)

Explainable artificial intelligence (XAI) is a number of techniques and methods with the goal of making artificial intelligence models decision-making processes more clear, interpretable, and comprehensible to humans. Increasing complexity and prevalence of AI systems, particularly with deep learning models, has led to raise concerns regarding trust, accountability, and ethical usage in the future about the Blackbox appearance of AI models [25]. XAI has been conceptualized to provide insights into how models arrive at their decisions and predictions, allowing the end-user to understand the underlying logics, identify possible biases, and test the authenticity of AI-based decisions. This is particularly crucial in high-stake areas such as health, finance, and security, where understanding the rationale behind AI decisions is of paramount importance for fairness, safety, and compliance adjacent to regulations. Several clinical case studies show the usefulness of SHAP and LIME, which offer complementary ways to model interpretability in clinical contexts such as seizure detection [11], Parkinson diagnosis [12], diabetes diagnosis [13], and heart disease prediction [26].

Although SHAP (SHapley Additive exPlanations) and LIME (local interpretable model-agnostic explanations) belong to the most widely used post hoc explainer techniques in explainable AI (XAI), both are trained to explain the predictions of complex, machine learning models. SHAP utilizes Shapley values, which fairly distribute the prediction outcome among all input features by considering all possible feature combinations. This ensures a consistent and mathematically grounded explanation of the model’s decision-making process. SHAP provides both global interpretability, which helps understand the overall impact of features on the model’s predictions, and local interpretability, which explains individual predictions by identifying the most influential features for a specific instance. However, LIME is different, though, as it works regarding learning local behavior by using a less complex, understandable model (like linear regression) to approximate the behavior of a rather complex model around a certain point of interest. Data were represented in a manner that constructs perturbations of the input data around a certain instance of interest, measuring the effect on predictions by the complex model. Then, it locally approximated the behavior of the model in the neighborhood using a simple interpretable surrogate model. By using this surrogate model, it can assess which features were the most important in getting to its prediction for that specific data point and give insight into the model’s decision. This method enables users to understand why the model made a certain decision, especially in black-box models like neural networks and ensemble methods. These methods generate explanations that are intuitive and understandable to humans, so the stakeholders recognize how individual predictions are being made, which is of utmost importance to clinical applications such as CKD diagnosis, where interpretability and trust are of utmost importance.

A method for explaining machine learning models’ predictions, especially those of deep neural network, is called integrated gradients. By integrating the gradients of the model’s output with respect to the input features along a path from a baseline input to the actual input, it gives each input feature a relevance score. Any differentiable model can be used with this approach because it is model agnostic.

## 4. Proposed CKD Prediction for MIoT

**Figure 4:**
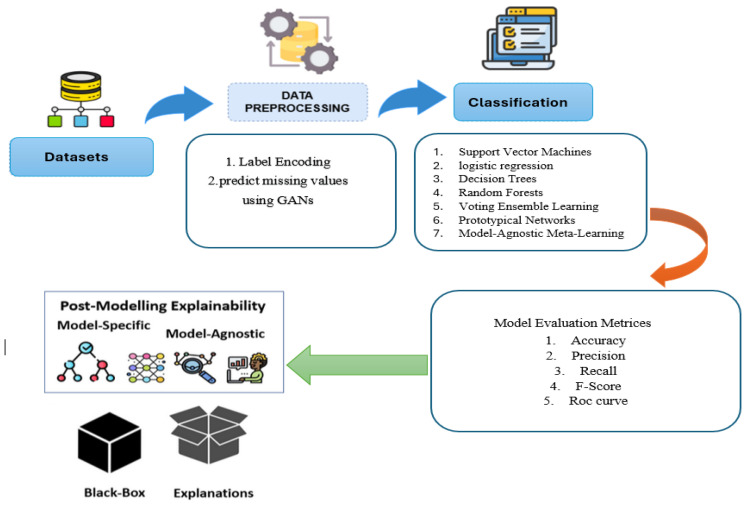
Proposed CKD prediction system.

This section outlines the key stages of our proposed CKD prediction model and illustrates its development for managing the complex data commonly encountered in MIoT applications. Figure 4 illustrates the systematic approach used in this study for CKD prediction, comprising five key steps: data collection and storage, data preprocessing, machine learning model training, model evaluation and validation, and explainable artificial intelligence (XAI) for model interpretation. Each step is detailed as follows:

Step 1: Data Collection and Storage: The diagnostic data were gathered from medical records, traditionally collected by healthcare professionals. Then categorical variables were converted into numerical values using label encoding. The dataset was analyzed for missing values, which were later imputed using generative adversarial networks (GANs) [27].

Step 2: Data Preprocessing: Generative adversarial networks (GANs) were employed to handle missing data by: establishing a mask for missing values and preprocessing the dataset. A generator and discriminator are designed for the dataset. The GAN is then trained adversarial to learn the data distribution. The trained generator is used to fill in missing values, and the imputed values are evaluated and post-processed.

Step 3: Machine Learning Model Training: Seven different machine learning models were used to predict CKD. Traditional models include support vector machines (SVMs), logistic regression (LR), decision trees (DT), random forests (RF), and voting ensemble learning (VEL). Additionally, few-shot learning techniques such as prototypical networks and model-agnostic meta-learning (MAML) [28] were employed to enhance performance in limited data scenarios.

Step 4: Model Evaluation and Validation: The trained models were evaluated using accuracy, precision, recall, F1-score, confusion matrix, and area under the receiver operating characteristic curve (ROC-AUC) to assess predictive performance. Internal validation was conducted on CKD datasets to ensure model robustness. To further enhance generalization, few-shot learning techniques were used in cases with limited data availability.

Step 5: Explainable Artificial Intelligence (XAI) for Model Interpretation: To enhance interpretability, explainable artificial intelligence (XAI) techniques were applied:

1. SHapley Additive exPlanations (SHAP): Provided both global and local interpretability based on game theory. It explained the contribution of each feature to the model’s predictions.
2. Local interpretable model-agnostic explanations (LIME): Assessed how individual input features influenced predictions and identified even minor feature effects on CKD prediction [29].

## 5. Experimental Results

This section provides an overview of the CKD dataset, along with the performance metrics and a detailed analysis of the proposed system’s results.

### 5.1. Dataset

**Figure 5:**
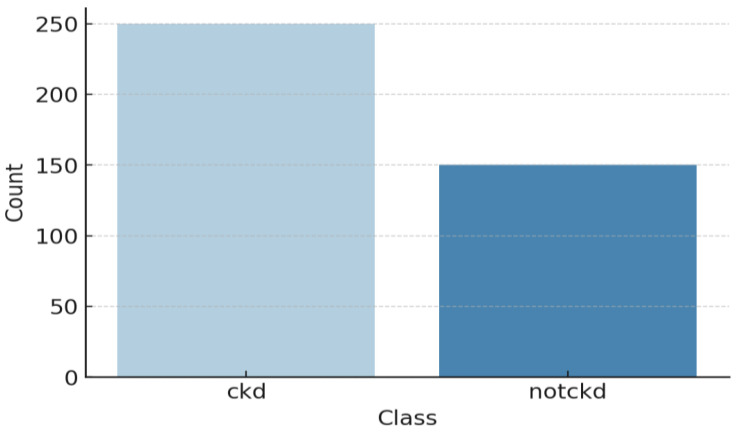
Class distribution of the CKD dataset.

**Figure 6:**
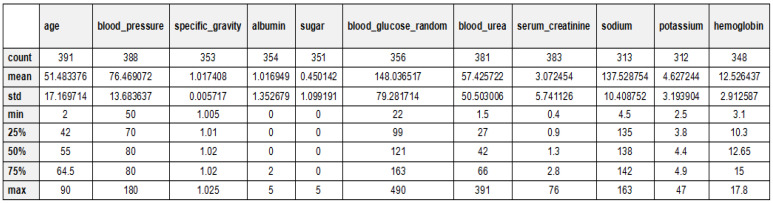
Statistical analysis explanation of the CKD dataset.

Table 2 and Figure 5 provide an overview of the dataset used for chronic kidney disease (CKD) classification, highlighting the distribution of cases across two categories. The dataset consists of 250 CKD cases, representing patients diagnosed with chronic kidney disease, and 150 non-CKD cases, referring to individuals without CKD. This distribution indicates a slight class imbalance, which should be considered when training machine learning models to prevent bias toward the majority class. Techniques such as data balancing (e.g., SMOTE for oversampling) or cost-sensitive learning may be necessary to improve model performance and ensure fair predictions across both categories. The 24 features for CKD diagnosis have been documented in Table 3 along with their descriptions. These features are patients’ demographic data, laboratory tests, and other medical conditions, which contribute towards classifying CKD. Each of these critical parameters is important to evaluate every aspect of CKD to enhance the classification process. The statistical analysis of the CKD dataset is depicted in Figure 6.

### 5.2. Evaluation Metrics

In this work, the effectiveness of the proposed model for the diagnosis of chronic kidney disease (CKD) using accuracy, F1-score, precision, recall, and ROC curve, where the percentage of the test set that the classifier successfully classifies represents the accuracy of the classification model on that test. The precision of positively labeled examples is determined by their accuracy. Recall is a metric that quantifies how accurate or comprehensive positive examples are, that is, how many instances of the positive class have the proper label applied. Table 4 illustrates the confusion matrix parameters used for evaluating the model’s performance. These metrics are derived from classification outcomes and help in computing evaluation metrics such as accuracy, precision, recall, F1-score, and Matthews correlation coefficient (MCC) [30,31]:

(1) $$\mathrm{Accuracy}=\frac{TP+TN}{TP+FP+FN+TN}$$

(2) $$\mathrm{Precision}=\frac{TP}{TP+FP}$$

(3) $$\mathrm{Recall}=\frac{TP}{TP+FN}$$

(4) $$F1\text{-}\mathrm{Score}=2\{\frac{Precision*Recall}{Precision+Recall}\}$$

(5) $$\mathrm{MCC}=\frac{\left(TP\times TN\right)-(FP\times FN)}{\sqrt{\left(TP+FP)(TP+FN)(TN+FP)(TN+FN\right)}}$$

where (*TN*) true negatives, (*TP*) true positives, (*FP*) false positives, and (*FN*) false negatives.

### 5.3. Results Analysis

**Figure 7:**
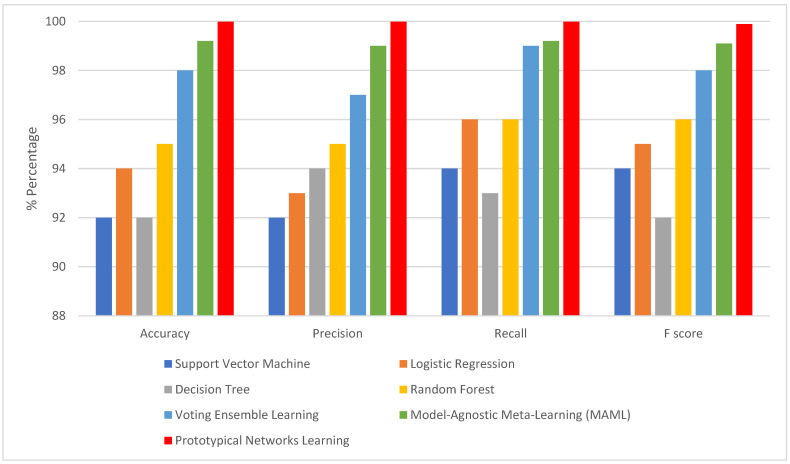
The matrices when handling missing using GAN.

**Figure 8:**
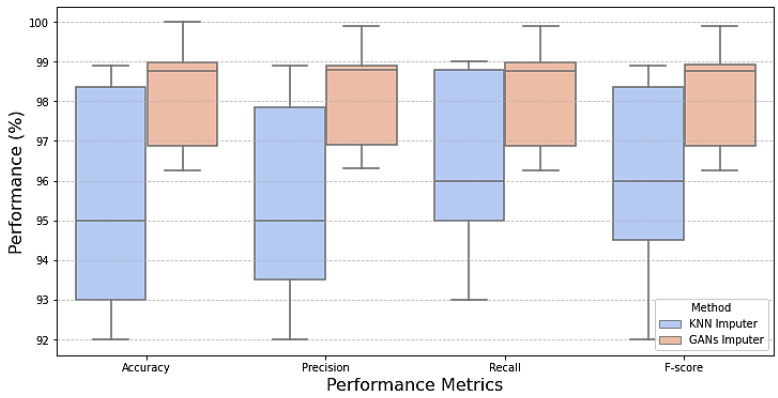
Box plot comparison of model performance for missing data handling.

Examining model performance is essential to see how each algorithm performs in classification during testing and training. The model learns the hidden patterns in the data during training, and then it uses the unseen data to make predictions during testing. Seventy percent of the data was used to train the model. The optimized models were tested using the remaining 20%. After each model was optimized, we compared seven different models to assess how well they performed. As shown in Table 5, KNN imputation gives good results. This evaluation considered several parameters, including recall, accuracy, precision, and F1-score when dealing with missing values with traditional machine learning imputation using KNN imputation [32]. However, from Table 6 and Figure 7, it is shown that improvement in the results when dealing with missing values using GANs. It is revealed that the outperformance of both prototypical networks learning and MAML is all across all metrics. Prototypical networks learning is shown its efficacy in the learning phase by achieving high accuracy (99.99%) and outstanding precision (99.9%). Also, its recall score (99.2%) is good; prototypical networks few-shot learning achieved flawless training results. Like the prototypical networks few-shot learning model, the MAML model achieved good results. So, the few-shot learning models were chosen as the top-performing models after considering; this is especially significant in a CKD where a precise diagnosis is essential. High precision minimizes false positives, saving patients needless worry and additional testing, while high sensitivity (recall) is essential for guaranteeing that patients with CKD are accurately detected [33]. A box plot for the ML model performance with GAN imputation is illustrated in Figure 8.

**Figure 9:**
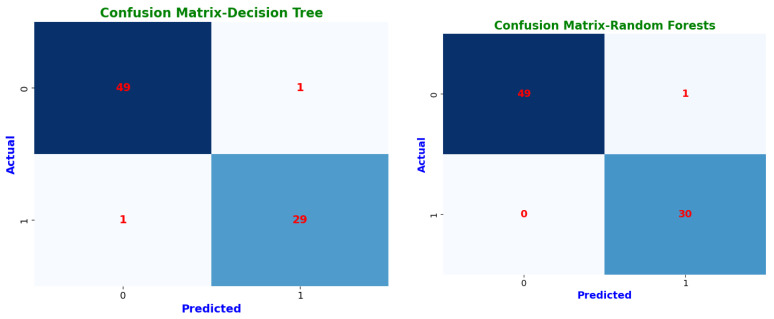

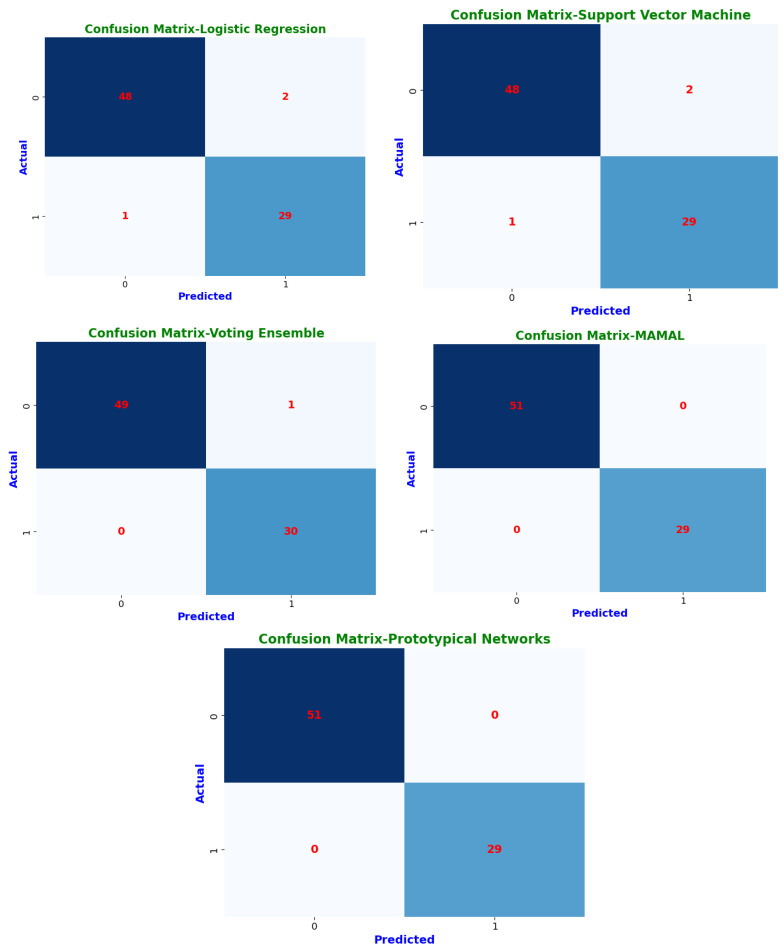
Confusion matrices of machine learning classifiers.

Confusion matrices highlight each model’s classification abilities in further depth (Figure 9). The classification results are sorted into four groups by these matrices. Instances where a CKD patient was accurately detected are known as true positives (*TP*). True negatives (*TN*) are instances in which a person without chronic kidney disease is correctly identified. False positives (*FP*) are instances in which a person was mistakenly diagnosed with chronic kidney disease (CKD) when they tested negative. Finally, cases where we mistakenly classified a person as non-CKD when they were truly positive are known as false negatives (*FN*) [34].

These confusion matrices help in understanding model performance and detecting possible misclassifications. Prototypical networks and MAML achieved perfect classification, correctly identifying all CKD (TP = 29) and non-CKD cases (TN = 51) with zero misclassifications (FP = 0, FN = 0). This resulted in an MCC of 1.0, indicating optimal classification performance. Random forest (RF) and voting ensemble models also exhibited exceptional accuracy, correctly classifying 49 non-CKD cases and 30 CKD cases, with only one misclassification (FP = 1, FN = 0). Both models attained an MCC of 1.0, reflecting high reliability and minimal error rates. The decision tree (DT) demonstrated strong classification performance, correctly predicting 49 non-CKD cases and 29 CKD cases, with a slight misclassification of one CKD case as non-CKD (FN = 1, FP = 1). Its MCC of 94.67% shows it remains a dependable classifier. Logistic regression (LR) and support vector machine (SVM) performed similarly, with 48 correct non-CKD classifications and 29 correct CKD predictions, but with two non-CKD cases misclassified as CKD (FP = 2). Their MCC scores of 94.73% confirm that they remain robust but slightly less precise than ensemble models.

**Figure 10:**
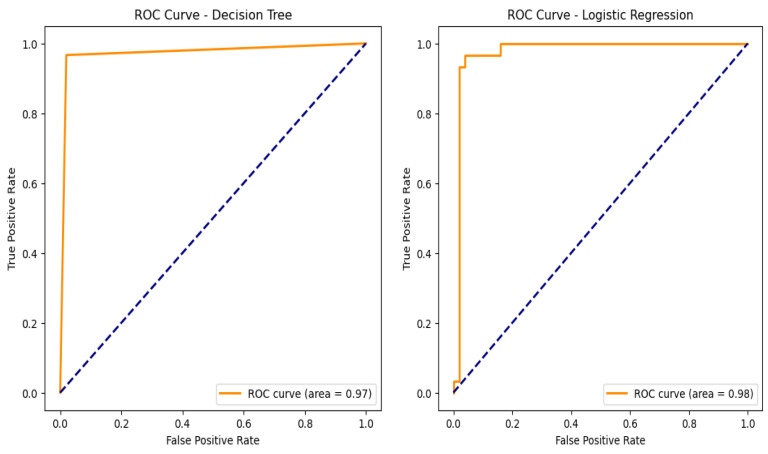

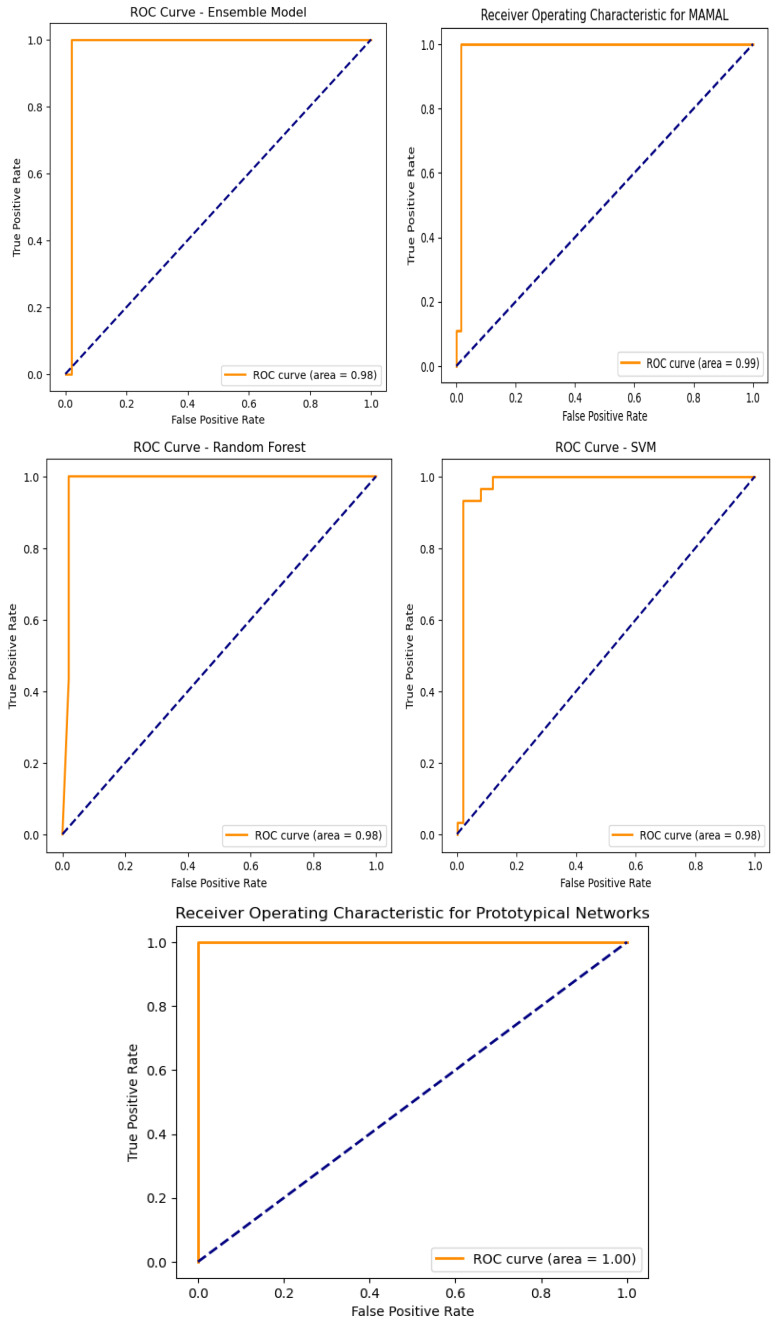
Machine learning model receiver operating curves (ROC).

An assessment of the trade-off between true positive and false positive rates can be made by looking at the ROC curves as shown in Figure 10 and the corresponding AUC values for the models used in the categorization of chronic renal disease. The prototypical networks, a few-shot learning model, and MAML achieved an exceptionally high AUC of 0.999, demonstrating outstanding generalization capability and superior classification performance. The decision tree (DT), with an AUC of 0.97, showed reliable classification performance, though slightly lower than other models. The support vector machine (SVM), logistic regression (LR), ensemble learning, and random forest (RF) models all achieved an AUC of 0.98, confirming their strong ability to accurately distinguish between CKD and non-CKD cases [33]. These results validate that few-shot learning models, particularly prototypical networks, provide the best classification performance.

**Figure 11:**
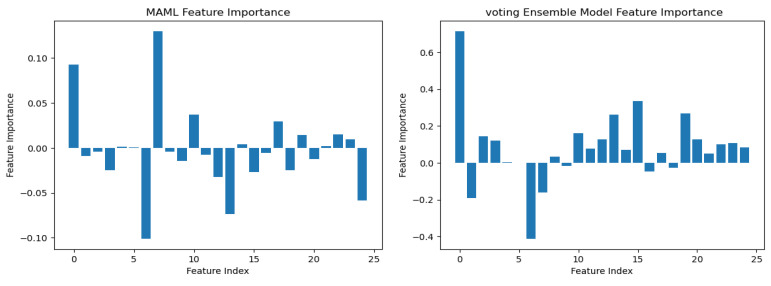

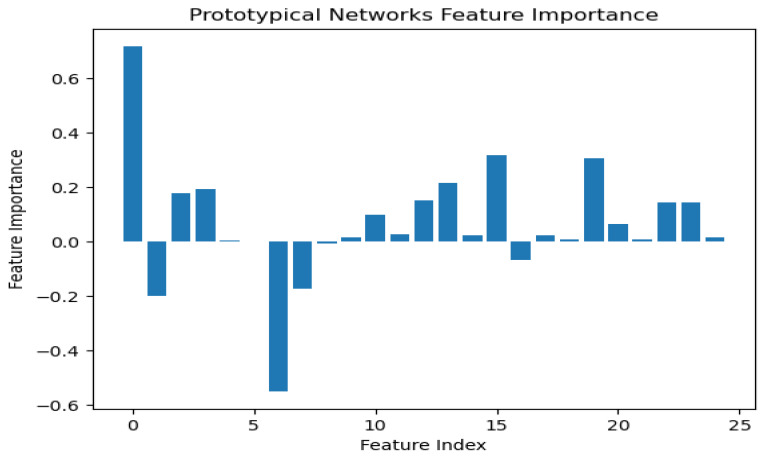
Integrated gradients summary plot for machine learning.

To understand the logic behind CKD predictions, the model explanations were interpreted using the best model, prototypical networks learning. The SHAP (SHapley Additive exPlanations) global explanation of CKD data is shown in Figure 11. Global explanations encompass the entire dataset.

**Figure 12:**
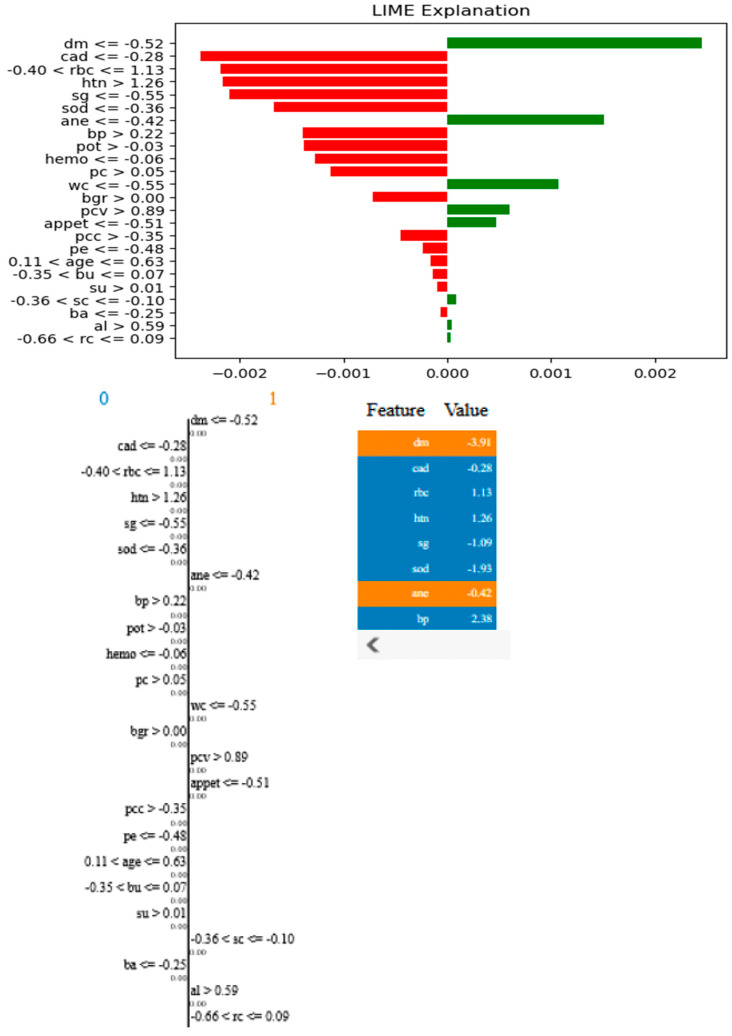
LIME plots of KCD features.

According to the findings of the performance analysis carried out using these statistical indices, prototypical networks learning is the most effective model for predicting the KCD. It consistently outperformed both MAML and other machine learning across all criteria. The prototypical networks few-shot learning model outperformed other machine learning models, and the underlying process of the outcomes it generated was examined using the SHAP explanation. Figure 11 displays the meaning of absolute SHAP values, or feature importance, for the prototypical networks model. This figure assesses how interpretable for MAML, a voting ensemble, and prototypical networks. MAML and prototypical networks have more balanced feature importance distributions and more structure to them, while the voting ensemble model seems more scattered. Prototypical networks effectively point out the key features that impact prediction within a narrower scope that guarantees that the model predicts based on the most relevant information. MAML, on the other hand, attributes importance to a wider spectrum of features while retaining stability, which speaks of its generalization capacity in different scenarios. In contrast, the voting ensemble model seems to give tremendous importance to a few of the features, neglecting others, resulting in overfitting and limited adaptability to the real world. Therefore, the approaches pursued by MAML and prototypical networks are seen as much more trustworthy and interpretable, as they could be seen to enhance robustness or relevance in feature selection. LIME is a potent XAI technique that may be used to comprehend the intricate correlations between KCD measures and their influence on overall kidney potability since it approximates a complex machine learning model with a simpler, interpretable model. In terms of KCD, this implies that LIME can help determine which KCD criteria are most important in predicting a particular KCD. The model predicted whether the kidney was diseased or not because of these characteristics. Researchers and decision-makers can benefit greatly from this information since it helps pinpoint the specific issues that must be resolved to improve the KCD prediction, as illustrated in Figure 12.

From the figure above, it is shown that the most relevant features will have the strongest effects, such as “dm ≤ −0.52” (diabetes mellitus), which has the most positive effect, and “cad ≤ −0.28” (coronary artery disease), which has the most negative effect. Other important features include “htn > 1.26” (hypertension) and “bp > −0.02” (blood pressure), indicating their significant role in the model’s decision making. The LIME method decomposes individual predictions to facilitate the understanding of black-box models, ensuring transparency and interpretability in AI-based CKD detection.

The study’s findings highlight how well the prototypical networks GANs model and explainable artificial intelligence (XAI) work together to diagnose chronic kidney disease (CKD). Our study confirms prototypical networks’ applicability in the setting of CKD, which is consistent with earlier research showing its accuracy and efficiency in many medical scenarios. The current study, however, fills a critical gap in the field of medical AI where understanding the rationale behind the model is just as important as the diagnostic outcome. It goes beyond simply concentrating on diagnostic accuracy and emphasizes the importance of model interpretability utilizing XAI. This study has noteworthy practical benefits, particularly in kidney care. This work demonstrates the reliability of explainable machine learning in diagnosing CKD, which may pave the way for the eventual integration of these technologies into routine clinical practice. This combination could lead to a faster and more accurate diagnosis of CKD, enabling prompt action to improve patient outcomes and reduce the progression of the illness. Physicians will be using SHAP and LIME in CKD diagnoses by interpretable insights into machine learning modeling. By allowing importance to be laid on serum creatinine, diabetes mellitus, and proteinuria, SHAP serves to help hospitals in validating the prediction by an AI model. By explaining a diagnosis as due to certain symptoms, such as high blood pressure or diabetes history, LIME elucidates individual diagnoses that contribute to a patient’s CKD classification. Besides identifying indicators of deterioration in hemoglobin levels, SHAP could provide predictions on the progression of CKD, enabling early intervention in most cases. SHAP could also identify biases in such AI models, thereby offering itself to unbiased risk assessment across different demographics.

The comparison with previous studies shows that the proposed technique provides outperformance against all other methods in the literatures as shown in Table 7.

The encouraging results of this study point to the need for additional research, particularly to examine the model’s performance in a range of clinical settings and patient types. To ensure the model’s adaptability and growth potential, future studies should concentrate on validating its performance through multicenter trials encompassing a broad range of clinical and demographic parameters. Furthermore, incorporating social determinants of health and genetic markers may improve the model’s predictive power and provide a more all-encompassing strategy for CKD management. The goal is to seamlessly incorporate AI-powered solutions into healthcare systems, revolutionizing the management of chronic kidney disease through models of customized, predictive, and preventative care.

### 5.4. Limitations and Challenges

Despite the promising outcomes of our study, several limitations and challenges must be addressed for broader applicability and real-world deployment of the proposed CKD prediction model:

▪ Generalizability to Diverse Populations—While the models demonstrated high predictive accuracy on the CKD dataset, their generalizability remains a concern. Additional validation on larger and more diverse multi-ethnic populations is necessary to ensure consistent performance across different demographic groups and healthcare settings.
▪ Reliance on High-Quality Data: The study relies on regularly collected pathological data, which may not always be available or standardized across medical institutions. Variability in data collection methods and missing values in real-world datasets could impact model performance.
▪ Dependence on GANs for Imputation: Although GANs were effective in handling missing data, their imputation process may introduce bias or synthetic artifacts that could influence prediction outcomes. As it basically learns from the existing data, any imbalance or inaccuracy in the original dataset will amplify in the generated samples. So, if the training dataset itself contains biases, there will be a problem in the generated data. So, a further validation is needed to assess the reliability of these imputed values across different datasets.
▪ Computational Complexity and Resource Requirements: The integration of advanced algorithms such as prototypical networks, model-agnostic meta-learning (MAML), SHAP, and LIME introduces computational overhead. Hence, this renders the analysis in real time very hard with highly complex deep neural networks. Real-time deployment in resource-constrained environments, such as small clinics or remote healthcare settings, may be challenging.
▪ Model Interpretability vs. Complexity Trade-off: While explainable AI techniques such as SHAP and LIME enhance model transparency, deploying these techniques in hospitals faces challenges, including computational cost, as it requires evaluating models many times for the generation of their explanations. Doctor training is also required because a majority of these clinicians do not understand any AI interpretability tools; hence, dedicated workshops need to be developed to understand the SHAP and LIME outputs in a clinical context. Compliance with all the healthcare regulations is another challenge, as AI has been proved stringent with data privacy regulations that must be met. They also require that hospitals ensure that they will not produce any biases or misinterpretations that might leak into the patient care process. Further research is required to develop more efficient interpretability frameworks for clinical decision making.
▪ Adaptability to Different Clinical Contexts: The model was primarily tested on CKD-related pathological data, and its adaptability to other types of medical data or healthcare conditions remains uncertain. Future studies should explore its applicability in different clinical scenarios to enhance its versatility.
▪ Ethical and Regulatory Considerations: The use of AI in healthcare raises ethical concerns regarding patient privacy, data security, and regulatory compliance. Confidentiality of the patient and data security are fundamental issues requiring the strict adherence of regulations such as HIPAA and GDPR in order to secure sensitive medical records from breaches and unauthorized access. In the case of AI-enabled CKD diagnosis, medical accountability and liability standards must also be followed. In cases where AI models make incorrect predictions, however, it is still ambiguous who can be held responsible, either the developers or the healthcare institutions that release the technology or the physicians themselves relying on it. To address this, regulations must define clear guidelines on AI-assisted decision making, ensuring that human oversight remains integral to the diagnostic process.
▪ Potential Bias in Feature Selection: The model heavily relies on specific clinical variables such as age and gender. However, other potential risk factors not included in the dataset might influence CKD progression. Further research should incorporate additional biomarkers and lifestyle factors to enhance predictive accuracy.

Addressing these limitations will be crucial for refining the proposed model and ensuring its effective deployment in real-world medical applications.

## 6. Conclusions and Future Work

Our study has effectively established explainable AI models that leverage routinely collected pathological data to accurately predict chronic kidney disease (CKD). The model utilizes a GAN to address missing values in CKD datasets and integrates few-shot learning techniques, including prototypical networks and MAML, with explainable machine learning for CKD prediction. By using vital parameters such as age, gender, and other crucial aspects of predicted CKD, this model showed significant prediction accuracy, especially when determining the probability of progression to kidney failure. Experimental results on CKD datasets demonstrated high performance, with prototypical networks and MAML achieving ROC-AUC values of 0.999 and 0.992, respectively, highlighting their strong predictive capabilities and potential applicability across diverse populations. It is shown that the key features align with the core pathophysiology of chronic kidney disease, enhancing the clinical significance of the developed models. Additionally, explainable AI such as SHAP and LIME improved model interpretability by offering clear, data-driven insights into prediction behavior at both local and global levels, enhancing transparency and trust. The study’s results emphasize the potential of precise predictive modeling in identifying high-risk CKD patients for personalized disease management in the framework of medical IoT. Future studies could explore the adaptability of the proposed approach across diverse data types and clinical settings to validate its effectiveness and broaden its applicability. Additionally, further validation in larger, multi-ethnic populations is necessary to enhance generalizability.

## Figures and Tables

**Table 1:** Summary of related work.

Work	AI Method	Merit	Demerit	Handling Missing Values
[13]	DeepNetX2 (explainable deep learning)	High accuracy in diabetes diagnosis; explainable AI improves transparency	Requires large datasets; computationally expensive	Not explicitly mentioned; likely uses imputation or exclusion of incomplete sample
[14]	Artificial intelligence (AI) for diagnosing diabetes complications	Effective in early detection of complications; scalable	Limited generalizability; depends on quality of input data	Imputation methods (e.g., mean, median, or k-NN imputation)
[15]	Machine learning (ML) with smart web application for CKD prediction	User-friendly interface; high prediction accuracy	Web application dependency; potential privacy concerns	Data preprocessing with imputation techniques (e.g., mean/mode imputation)
[16]	Comprehensive ML models for CKD prediction	High predictive power; robust performance across datasets	Complex model interpretation requires extensive computational resources	Advanced imputation methods (e.g., MICE or model-based imputation)
[17]	Interpretable ML for CKD progression risk prediction	Interpretable results; useful for clinical decision making	Limited to specific patient populations; may require frequent retraining	Handling missing data through imputation or exclusion based on clinical relevance
[18]	Comparison of ML techniques for early-stage CKD detection	Identifies best-performing models for early detection	Performance varies across datasets; may overfit on small datasets	Simple imputation methods (e.g., mean, median) or removal of incomplete samples
[2]	ML-based interface with explainable AI (XAI) for CKD diagnosis	Enhances trust in AI predictions; improves diagnostic accuracy	High implementation complexity; requires expert knowledge	Advanced techniques like multiple imputation or model-based approaches

**Table 2:** Dataset description.

Class Type	Classes Description	Number of Cases
CKD	Diagnosis CKD	250
Not CKD	Normal	150

**Table 3:** Description of chronic kidney disease features.

Features Names	Description	Features Names	Description
Age	Age patientage	HEMO	Hemoglobin
BP	Blood pressure	PCV	Packedcell volume
SG	Specificgravity	WC	White blood cell count
AL	Albumin	RC	Red blood cell count
SU	Sugar	HTN	Hypertension
RBC	Red blood cells	DM	Diabetes mellitus
PC	Pus cell	CAD	Coronary artery disease
PCC	Pus cell clumps	APPET	Appetite
BA	Bacteria	PE	Pedaledema
BGR	Blood glucose random	ANE	Anemia
BU	Blood urea	CLASS	Diagnosisckd, not CKD
SC	Serum creatinine		
POT	Potassium		

**Table 4:** The parameters of confusion matrix.

	Predicted CKD	Predicted Not CKD
Actual CKD	*TP*	*FP*
Actual Not CKD	*FN*	*TN*

**Table 5:** Table results algorithms after handling missing values using KNN imputer.

Algorithms	Accuracy	Precision	Recall	F Score
SVM	92.00	92	94	94
Logistic Regression	94.00	93	96	95
DT	92.00	94	93	92
RF	95.00	95	96	96
Voting Ensemble Learning	98.00	97	99	98
Prototypical networks few-shot learning	98.90	98.9	98.9	98.9
Model-agnostic meta-learning (MAML)	98.70	0.987	98.7	98.7

**Table 6:** Models’ performance when handling missing using GANs imputation.

Algorithms	Accuracy	Precision	Recall	F1-Score	MCC
Support vector machine	92	92	94	94	94.73
Logistic regression	94	93	96	95	94.73
Decision tree	92	94	93	92	94.67
Random forest	95	95	96	96	1
Voting ensemble learning	98	97	99	98	1
Model-agnostic meta-learning (MAML)	99.2	99.0	99.2	99.1	1
Prototypical networks learning	99.99	99.9	99.9	99.89	1

**Table 7:** Comparison of the proposed model to previous studies based on various matrices.

Technique	Accuracy	Precision	Recall	F1-Score
Ensemble deep learning, no feature selection [35]	98%	97%	99%	98%
Ensemble deep learning proposed hybrid with feature selection [35]	99.0%	85%	99%	92%
XgBoost classifier [21]	98.3%	98%	98%	98%
Support vector machine (SVM) with SMOTE [36]	99.33%	99%	99%	99%
Linear regression with tuning parameter [37]	99.36%	100%	99%	99%
Proposed model	99.99%	99.9%	99.9%	99.89%

## Data Availability

The original contributions presented in the study are included in the article; further inquiries can be directed to the corresponding author.
